# Musical intervention on anxiety and vital parameters of chronic renal patients: a randomized clinical trial

**DOI:** 10.1590/1518-8345.2123.2978

**Published:** 2018-03-08

**Authors:** Geórgia Alcântara Alencar Melo, Andrea Bezerra Rodrigues, Mariana Alves Firmeza, Alex Sandro de Moura Grangeiro, Patrícia Peres de Oliveira, Joselany Áfio Caetano

**Affiliations:** 1 Doctoral student, Departamento de Enfermagem, Universidade Federal do Ceará, Fortaleza, CE, Brazil.; 2 PhD, Adjunct Professor, Departamento de Enfermagem, Universidade Federal do Ceará, Fortaleza, CE, Brazil.; 3 RN.; 4 Doctoral student, Universidade Federal da Paraíba, João Pessoa, PB, Brasil.; 5 PhD, Adjunct Professor, Departamento de Enfermagem, Universidade Federal de São João del Rei, São João del Rei, MG, Brazil.; 6 PhD, Associate Professor, Departamento de Enfermagem, Universidade Federal do Ceará, Fortaleza, CE, Brazil.

**Keywords:** Renal Dialysis, Anxiety, Complementary Therapies, Music Therapy, Clinical Trial, Nursing

## Abstract

**Objective:**

to evaluate the therapeutic effect of music on anxiety and vital parameters in patients with chronic kidney disease when compared to patients receiving conventional care in hemodialysis clinics.

**Method:**

randomized clinical trial conducted in three renal replacement therapy clinics. Sixty people with chronic kidney disease undergoing hemodialysis were randomly allocated to an experimental group and a control group, 30 persons per group). State anxiety was evaluated in both groups by the State-Trait Anxiety Inventory. A t-test was used to verify the effect of the experimental manipulation on the variables.

**Results:**

we found a statistically significant difference between the groups regarding the degree of anxiety experienced during hemodialysis sessions. The experimental group presented a statistically significant reduction of anxiety scores (p = 0.03), systolic blood pressure (p < 0.002), diastolic blood pressure (p < 0.002), heart rate (p < 0.01) and respiratory rate (p < 0.006) after listening to music.

**Conclusion:**

music represents a potential resource for nursing intervention to reduce state anxiety during hemodialysis sessions. Brazilian Registry of Clinical Trials: RBR-64b7x7.

## Introduction

Chronic kidney disease (CKD) is considered a major global public health problem. It begins as a kidney injury that leads to the progressive functional loss of this organ, in which glomerular filtration rates drop to less than 15 ml/min. In its final stage, this condition requires some form of renal replacement therapy, with hemodialysis being the most commonly used treatment modality^1^ ^-^ ^2^. It is estimated that 10% of the world’s population is affected by CKD^3^ ^-^ ^4^ and in Brazil, it is believed that two million Brazilians have some degree of renal dysfunction^1^.

CKD is a traumatic disease with significant psychic consequences to the patient’s life. The whole course of treatment is experienced as an arduous and fraught experience that entails different physical, social and emotional limitations. These experiences cause significant changes in family life, as well as loss of autonomy and dependence on the Social Security System^1^ ^,^ ^4^ ^-^ ^6^.

The limitations caused by CKD make mental disorders such as depression^4^, stress^5^ and anxiety^4^ ^-^ ^5^ ^,^ ^7^ frequent in dialysis patients. Anxiety is defined as unpleasant mental feelings, preoccupation and tension associated with physical symptoms such as agitation, headache and palpitations^8^.

 Anxiety and stress have an overwhelming effect on individuals undergoing hemodialysis, causing increase in mortality, frequency of hospitalizations and treatment costs^7^. It also precludes changes in lifestyle, adherence to diet and to recommended therapies and deterioration in *performance status* ^9^, requiring urgent intervention by professionals who provide care for this clientele.

This study points out that, compared to stress and depression, anxiety has received little clinical attention, despite its high prevalence among CKD patients^4^. There are many strategies to ameliorate this symptom, among which are non-pharmacological interventions such as auricular therapy, systemic acupuncture and music therapy. The latter is an intervention recommended by the *Nursing Interventions Classification* (NIC) (4400) and it is defined as the use of music to help achieving a specific change in behavior, feeling or physiology of patients^10^.

Some measures proposed by the NIC to be taken at the moment of performing music therapy include: defining the desired specific behavior and/or physiological change (relaxation, stimulation, concentration, pain reduction); informing the individuals about the purpose of the musical experience; electing music selections that are particularly representative of the individuals’ preferences; helping the individuals to adopt a comfortable position; limiting external stimuli (e.g. lights, sounds, visitors, phone calls) during the listening experience; providing earphones, if convenient; and ensuring adequate volume, among other activities^10^.

Music has been identified as a complementary therapeutic resource in the nursing practice, for management and control of signs and symptoms, as well as in the patient-nurse communication and relationship, making care more humanized^11^ ^-^ ^13^. Research claims that the physiological effects of music involve sensorial, hormonal and physiological-motor reactions, such as metabolic changes, adrenaline release, respiratory rate regulation, blood pressure changes, reduction of fatigue and muscle tone, increase of threshold sensory stimuli, and improvement of attention and concentration^12^ ^-^ ^14^. It is also an excellent therapeutic tool, easy to use, accessible, without side effects and applicable in several contexts and for several diseases^12^ ^,^ ^15^ ^-^ ^18^.

This study is justified by the fact that research in this area, focusing on the effect of music therapy on reduction of anxiety and vital parameters of people undergoing hemodialysis, are scarce until present date^19^, and we believed that nurses play an important role in the care of CKD patients undergoing hemodialysis treatment, since they constitute the axis that brings together a series of interdisciplinary actions. The purpose of this study was to evaluate the therapeutic effect of music on anxiety and vital parameters in patients with chronic kidney disease when compared to patients receiving conventional care in hemodialysis clinics.

## Method

This is a randomized controlled clinical trial performed in three renal replacement therapy clinics in the state of Paraíba, Brazil, with 60 CKD clients on hemodialysis treatment. The study was developed between May and July 2016.

The inclusion criteria were: patients aged 18 years or more, literate, with a Glasgow scale score of 15, and presenting preserved auditory acuity as assessed by propaedeutic auditory tests (tuning fork tests of Weber and Rinne). The exclusion criteria were: use of anxiolytics within a period of up to 24 hours before the music intervention, presence of a history of psychiatric illness and hemodynamic instability.

**Figure 1:**
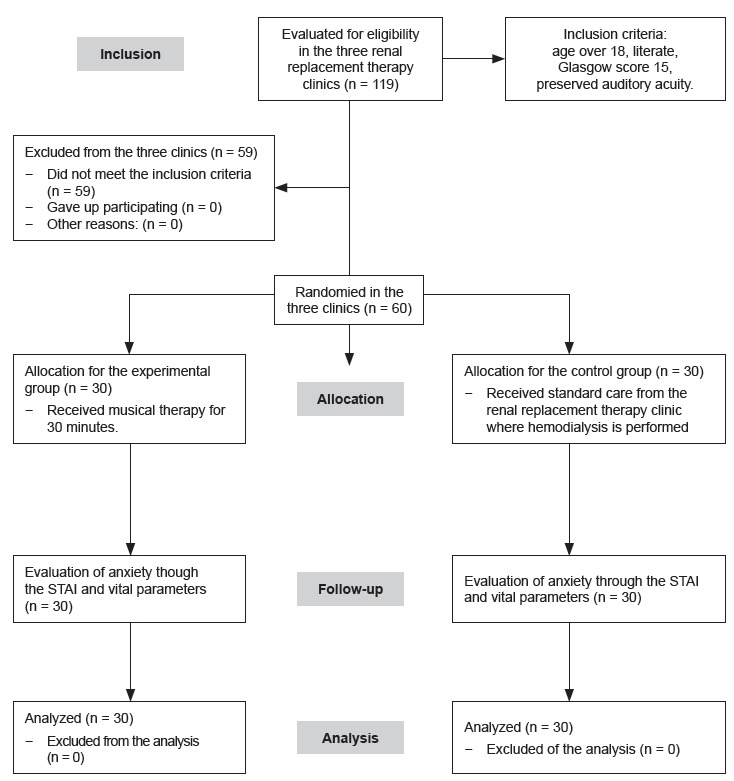
Flowchart of the study. Cajazeiras, Guarabira, Patos; Paraíba; Brazil, 2016

The sample size was calculated for both groups, assuming a significance level of 5% and test power of 80%, considering a minimum difference to be detected of five points in the outcome variable. Thus, a sample size of 30 CKD clients on hemodialysis treatment was obtained in each group. Figure 1 shows the flowchart of the participants who received the intended treatment and who were analyzed for the primary outcome. Among 119 participants assessed for eligibility, 59 were excluded because they did not meet the inclusion criteria. Among them, 20 patients were from in the clinic 1, 27 from the clinic 2 and 12 from the clinic 3. The reasons were: clinic 1: 12 because they were illiterate, seven for having used anxiolytic drugs in the last 24 hours and one for having a history of psychiatric illness; clinic 2: 20 because they were illiterate, 7 for having used anxiolytic drugs in the last 24 hours; and clinic 3: 10because they were illiterate, two for having used anxiolytic drugs in the last 24 hours. The sixty patients were randomly allocated either to an experimental group (n = 30) and a control group (n = 30). There were no losses in the follow-up or analysis (Figure 1).

Randomization was performed using the random number table generated in thr Epi Info *software* version 7.1.4, in two groups: experimental group (EG) and control group (CG), with a 1: 1 allocation rate, by a professional who had no contact with the researchers. Sixty envelopes were sequentially numbered with the label “experimental group” or “control group”. The sealed envelopes were delivered to the principal investigator.

In order to ensure the blindness of the allocation of participants until the intervention, the nurse in the service selected the patients that met the inclusion criteria. With the list of eligible patients in the hands, the researcher addressed the patient who was already connected to the hemodialysis machine, in the first 30 minutes of the therapy, to measure the baseline levels of the outcome variables (anxiety and vital parameters) and to collect sociodemographic and clinical data. It should be noted that in order to guarantee the concealment of the allocation of participants, their assignation was only known to the assistant researcher after opening the properly sealed envelope, which informed the condition selected for that participant. Only at this time, patients were allocated either to one of the two groups. The envelopes were sequentially used in order of numbering from 1 to 60. Based on this procedure and after the assignation of participants to the CG and EG, the research stages, i.e. measurement of vital signs, application of instruments, and application of the music therapy to the EG, were carried out. After the experimental manipulation, a second measurement (retest) was carried out, in which the abovementioned measures were taken a second time. Not only the researcher but also the person responsible for the statistical analyses was also blinded; for this, the CG and EG were coded as G1 and G2 before the data were made available in order to prevent the statistical researcher to know which group had received the intervention.

Two instruments were used to collect data: a questionnaire for sociodemographic and clinical data (age, marital status, educational level, family income, presence of caregiver, spiritual support, type of venous access for hemodialysis, presence of pain, heart rate, systolic blood pressure, diastolic blood pressure, and respiratory rate); and the *Stait-Trait Anxiety Inventory (*STAI), which is one of the most commonly used measures of self-reported anxiety in research and clinical practice in different cultures^20^. This scale has been translated and adapted for Brazil in 1979, with psychometric measures superior to the English and Spanish versions. Internal consistency showed that Cronbach’s alpha coefficient was 0.93 for men and 0.88 for women^21^.

The STAI instrument consists of two subscales (trait anxiety, which presents assertions for the individuals to describe how they usually feel, and state anxiety, which requires the individuals to describe how they feel at a given moment^20^ ^-^ ^22^, which in the present study corresponded to the moment of hemodialysis session). Each of these subscales is composed of 20 questions with a Likert-type response options, as follows: 1 - almost never; 2 - sometimes; 3 - often; 4 - almost always. The score varies from 20 to 80 points, with higher scores indicating higher levels of anxiety^22^, which may indicate trait of state anxiety in: low (0-34), moderate (35-49), high (50-64) and very high (65-80) grades. The state anxiety scale (20 questions) was used in the present study because the objective was to evaluate anxiety at the moment of dialysis and not to diagnose the tendency for anxiety.

Regarding the measurement of vital parameters, care was taken to improve the adequacy of the measurement, in which, before data collection, the researcher followed a manual with information for adequate execution of the procedures for measuring vital parameters. Regarding blood pressure measurement, we used the indirect method with auscultatory technique and calibrated aneroid sphygmomanometer (standardization).

The technique of verification and evaluation of blood pressure measures followed the protocol recommended by the Brazilian Society of Cardiology in the Brazilian Guidelines for Hypertension^23^. Heart rate was measured using radial palpation techniques, counted in one minute and estimated in beats per minute (bpm), and respiratory rate was determined according to the number of inspirations per minute (ipm).

Data collection occurred in the first 30 minutes of dialysis therapy. Data were collected in the following order: verification of vital parameters and application of the sociodemographic questionnaire and the STAI scale (pre- and post- intervention) by an assistant researcher; opening of the envelopes and allocation of the patient in one of the groups by a nurse from the service and intervention by the principal investigator.

The intervention was then initiated in the EG following the recommendations of the NIC^10^, including the definition of the desired physiological and behavioral change, which in the present study was relaxation and reduction of anxiety and vital parameters (Blood Pressure, Pulse and Respiratory Rate); explanation of the purpose of the musical experience to the participants; help to maintain the patient in a comfortable position; provision of earphones, ensuring proper volume and limiting external stimuli. In order to avoid external stimuli, the companion of the patients and health professionals were asked who not interact with the patients while they were listening to music, as well as to turn off cell phone devices during the experience of listening to music. Concerning the intervention proposed by the NIC to select music that were particularly representative of the preferences of the individuals, reference was made to previous research^12^ ^,^ ^24^, which affirms that soft classical songs present low amplitudes, simple and direct rhythms, and frequencies (time) of approximately 60 to 70 beats per minute, which are characteristics that promote relaxation, the intended goal of the present study. Thus, the classical song Spring from Vivaldi’s Four Seasons was chosen, as this song meets these prerequisites.

The musical reproduction was performed by means of individual headphones coupled to an MP3 player, for a period of 30 minutes, in the very armchair where the client was sitting for hemodialysis. The option for 30 minutes was based on other studies on the same subject with adult population^24^ ^-^ ^25^. The volume was controlled by the participant and during that time he had no contact with any health professional or companion.

It is noteworthy that the same time interval was observed for CG participants. In the case of this group, the main investigator performed the therapeutic intervention in the first and last five minutes of the 30-minute period, also at the beginning of dialysis therapy.

After the 30-minute interval, the outcome variables (anxiety and vital parameters) for were re-measured in both groups.

We emphasize that the intervention was applied to only one patient per dialysis shift. This was so for two reasons: the need to perform the intervention within the first 30 minutes of dialysis, because dialysis removes liquids from the patient and consequently reduces blood pressure; and to avoid that EG and CG patients had no contact with each other. In order to avoid sample contamination, collection took place at three different clinics. The three clinics are private institutions, yet all patients assisted there are forwarded by the Unified Health System (SUS). Thus, there was no patient covered by health insurance plans. The three clinics have the same service profile, with similar physical structure and human resources.

Data were processed through double typing to control possible errors in the *Statistical Package for Social Sciences* (SPSS) software, version 22.0. The SPSS was also used for the statistical analyses, adopting a significance level of 5%, corresponding to p < 0.05 (95% confidence interval). Descriptive statistics were used to obtain absolute distributions, percentages, means and standard deviations in order to present the studied variables, in addition to the *Pearson* correlation analysis. A t-test was used to verify the effect of music therapy on the studied variables.

In order to participate in the study, all participants received an Informed Consent form, and their confidentiality and anonymity was ensured. The development of the study met national and international standards of research ethics involving human subjects and was approved by the Research Ethics Committee of the Federal University of Ceará under number 1,482,535 and clinical record in the database of the Brazilian Registry of Clinical Trials with primary identifier: RBR-64b7x7.

## Results

Sixty CKD patients on hemodialysis participated in the study. The characterization data of the sample are shown in Table 1.

**Table 1:** Comparison of sociodemographic and clinical data between chronic renal patients on hemodialysis of the control and experimental groups before intervention. Cajazeiras, Guarabira, Patos; Paraíba; Brazil 2016

Sociodemographic and clinical variables		Control Group (n=30)		Experimental Group (n=30)		p-value*
Age in years		Mean 44,3	SD^†^ (13,9)	Mean 42,1	SD^†^ (13,4)	0,534^‡^
Gender		n	%	n	%	
	Female	14	46,7	13	43,3	0,795^§^
	Male	16	53,3	17	56,7	
Caregiver		n	%	n	%	
	Yes	18	60,0	16	53,3	0,397^§^
	No	12	40,0	14	46,7	
Education		n	%	n	%	
	Incomplete primary school	14	46,7	14	46,7	0,320^§^
	Complete primary school	1	3,3	5	16,7	
	Incomplete secondary school	4	13,3	1	3,3	
	Complete secondary school	9	30,0	8	26,6	
	Complete higher education	1	3,3	2	6,7	
	Postgraduate degree	1	3,3	0	0,0	
Marital status		n	%	n	%	
	Single	10	33,3	12	40,0	0,960^§^
	Married	18	60,0	16	53,3	
	Divorced	1	3,3	1	3,3	
	Widower	1	3,3	1	3,3	
Religion		n	%	n	%	
	Does not have	2	6,7	1	3,3	0,709^§^
	Catholic	23	76,7	22	73,3	
	Evangelical	5	16,7	7	23,3	
Family Income in Minimum Wages^||^		Mean	SD*	Mean	SD*	
	Less than 1 wage	1	3,3	1	3,3	0,355^§^
	1 to 3 wages	27	90,0	29	96,7	
	More than 3 wages	2	6,7	0	0,0	
Pain^¶^		n	%	n	%	
	No pain	30	100,0	28	93,3	0,150^§^
	Mild pain	0	0,0	2	6,7	
	Type of venous access	n	%	n	%	
	AVF**	26	86,7	26	86,7	1,000^§^
	DLC^††^	4	13,3	4	13,3	
State anxiety		n	%	n	%	
	Low	21	70,0	14	46,7	0,128^§^
	Moderate	8	26,7	14	46,7	
	High	1	3,3	2	6,7	
Vital Parameters		n	%	n	%	
	Systolic pressure in mmHg	147,9	25,8	152,9	28,0	0,475^‡^
	Diastolic pressure in mmHg	91,9	13,9	92,1	13,5	0,948^‡^
	Average beats per minute	78,6	12,5	82,4	13,7	0,266^‡^
	Inspiratory breath rate per minute	17,4	1,3	18,2	1,8	0,044^‡^

*p-value; †Standard deviation; ‡significance test (*p-value*) for the calculation of the t-test for independent samples; §significance test (*p-value*) referring to the chi-square calculation; ^||^minimum wage = $ 880.00 (US $ = 259.45); ¶Analog pain scale; ** Arteriovenous fistula; †† temporary double-lumen catheter.

Regarding state anxiety levels, the occurrence of low levels was more frequent in men (72.7%), followed by moderate levels (24.2%) and high levels (3.0%). Among women, moderate state anxiety (51.9%) was more frequent, followed by reports of low state anxiety (40.7%). The tests for comparison of means showed the statistical relevance of these differences; the mean score obtained by women was 38.1 (SD = 7.7) and, according to the t-test, state anxiety scale was significantly higher (*t* = 2.98, p = 0.004) than that observed in men, with a mean of 32.3 (SD = 7.4).

Another variable that had an effect on state anxiety levels was age. According to the linear correlation performed, a negative relation (linear correlation coefficient r = -0.26; p = 0.04) was detected between levels of state anxiety and the age of participants, which allowed us to infer that the older the patient, the lower the self-perceived state anxiety level. The presence of caregivers had no significant effect on state anxiety scores (*t* = -0.44; p > 0.05).

When the total scores of each participant (experimental group) were compared in the STAI state anxiety score before and after the intervention, there was a significant reduction (*t* = 2.24, p = 0.03) in the state anxiety reported by participants of the experimental group before (mean = 36.2, SD = 9.0) and after (mean = 32.8; SD = 9.6) the musical intervention. On the other hand, the participants in the control group did not present significant differences (*t*= 0.85, p > 0.05) between the mean scores obtained in the first evaluation, with a mean of 33.7 (SD = 6.7), and in the second evaluation, with an average of 33.3 (SD = 6.3), although the time elapsed between the measurements was equal in both the experimental and the control group.

In a more detailed way, 70.0% (n = 21) of the participants in the experimental group showed a reduction in perceived state anxiety level after musical intervention, while 6.6% (n = 2) did not indicate improvement and 23.4% (n = 7) reported a worsening in anxiety level. On the other hand, among the participants in the control group, it was observed that in the great majority of participants, the state anxiety levels reported worsened (46.7%, n = 14) or remained unchanged (23.3%, n = 7), while a reduction in state anxiety levels was observed in only 30.0% (n = 9) of the participants.

In general terms, an average reduction of 3.33 points was observed in the state anxiety scores reported by the participants submitted to the musical intervention, while the participants in the control group had an average reduction of 0.47 points.

In addition to evaluating the effect of musical intervention on self-perceived anxiety levels, we also sought to measure the effects of musical exposure on vital parameters (pulse, respiratory rate and blood pressure). The main results are summarized in Table 2.

**Table 2:** Comparison of anxiety and vital parameters of CKD clients on hemodialysis between the pre and post-intervention periods for the control and experimental groups. Cajazeiras, Guarabira, Patos; Paraíba; Brazil 2016

Parameter	Group	Pre-intervention			Post-intervention		Value p^†^
		Mean	SD*		Mean	SD*	
State anxiety level	Experimental (n=30)	36,2	9,0		32,8	9,6	0,033^‡^
	Control (n=30)	33,7	6,7		33,3	6,3	0,403^‡^
Blood pressure (Systolic) mmHg	Experimental (n=30)	152,9	28,0		139,9	24,9	0,002^‡^
	Control (n=30)	147,9	25,8		143,1	29,0	0,133^‡^
Blood pressure (Diastolic) mmHg	Experimental (n=30)	92,1	13,48		86,1	13,4	0,002^‡^
	Control (n=30)	91,9	13,9		86,2	13,1	0,020^‡^
Heart rate per minute	Experimental (n=30)	82,4	13,7		76,5	9,8	0,015^‡^
	Control (n=30)	78,6	12,5		82,2	14,1	0,167^‡^
Respiration rate in inspirations per minute	Experimental (n=30)	18,2	1,8		17,2	1,4	0,006^‡^
	Control (n=30)	17,4	1,3		17,5	1,5	0,600^‡^

*Standard deviation; †*p-value*; ‡significance test (*p-value*) for the calculation of the *t-*test for repeated measurements

The comparisons between groups showed that, in all parameters, with the exception of diastolic blood pressure, the mean value observed in the experimental group was higher than the control group in the pre-intervention period. This scenario reverses after the intervention, with a significant reduction of the parameters measured, where the participants submitted to the intervention present lower values than those observed for the participants in the control group.

A non-paired *t* test (Table 3) was performed to verify the statistical significance of this difference. The results showed that the reduction observed between the first and second applications in the state anxiety levels (*t* = 2.01; p = 0.048) in the participants who underwent musical intervention (experimental group) were significantly higher in the case of heart rate (*t* = 2.77, p = 0.007) and respiratory rate (*t* = 2.68; p = 0.01) when compared with participants not submitted to the intervention (control group). Regarding the parameters of systolic (*t* = 1.66, p > 0.05) and diastolic (*t* = 0.10, p > 0.05) blood pressure, no significant differences were observed between the reduction levels under the control and experimental conditions.

**Table 3:** Comparison between groups regarding the difference in anxiety levels and vital parameters in the pre- and post-intervention periods for the control and experimental conditions. Cajazeiras, Guarabira, Patos; Paraíba; Brazil 2016

Parameter	Group	M_difference_*	SD_difference_^†^	t (df)^‡^	p-value^§^	95% CI **	
						Inf	Sup
Nivel de Ansiedad Estado	Experimental (n = 30)	7,1	15,81	2,01 (58)	0,048^¶^	-15,87	-0,05
	Control (n = 30)	-0,7	14,78				
Presión Arterial (Sistólica)	Experimental (n = 30)	13,1	21,25	1,66 (58)	0,102^¶^	-18,22	1,68
	Control (n = 30)	4,8	17,02				
Presión Arterial (Diastólica)	Experimental (n = 30)	5,9	9,52	0,10 (58)	0,917^¶^	-6,07	5,47
	Control (n = 30)	5,7	12,59				
Pulso	Experimental (n = 30)	5,8	12,37	2,77 (58)	0,007^¶^	-16,24	-2,62
	Control (n = 30)	-3,6	13,93				
Frecuencia Respiratoria	Experimental (n = 30)	1,0	1,38	2,68 (58)	0,010^¶^	-1,98	-0,29
	Control (n = 30)	-0,1	1,86				

* Mean of observed differences between the first and second applications in the State Anxiety Scale scores; †Standard deviation of observed differences between the first and second applications in the State Anxiety Scale scores; ‡*t-*test value and degrees of freedom for non-paired measurements; *§p-value*; significance test (*p-value*) for calculation of the *t*-test for non-paired measures; ^**^95%Confidence Interval

## Discussion

Participants in both CG and EG groups had similar sociodemographic characteristics, as regards gender, presence of caregiver, age and type of venous access for hemodialysis. The majority of participants was male, had a caregiver, was aged on average 42.1 years (EG) and 44.3 years (CG) and had AVF as venous access. These findings are consistent with those already reported in the literature for patients on hemodialysis. The present study indicates the prevalence of men (63.07%), mean age of 49.7 years^26^ and fistula as predominant vascular access (93.8%)^27^.

As for average anxiety scores, most participants presented some degree of anxiety, of which 36.7% presented moderate anxiety. Authors believe that higher levels of anxiety in patients undergoing hemodialysis can be explained by the fact that they need to remain connected to the machine several hours a week, restricting their independence, not to mention the need to move to the clinics, keep a restricted diet and being unable to travel for prolonged periods of time^4^ ^-^ ^5^. As indicated by a randomized clinical study conducted with 54 CKD people undergoing hemodialysis, anxiety decreases the quality of life and may increase length of hospital stay of CKD patients^5^.

Thus, the results presented here give preliminary indications of the effectiveness of musical intervention in reducing the mean scores in state anxiety in patients undergoing hemodialysis. These findings are positive, since they point to the possibility of having a low cost intervention that allows greater well-being and quality of life for these patients. These results also agree with other studies performed with patients during invasive procedures that demonstrate that listening to music significantly relieves the perceived levels of anxiety^12^ ^,^ ^22^. Review and meta-analysis studies also complement by pointing out the effectiveness of musical intervention to reduce physiological and psychological stressors experienced by patients submitted to procedures in outpatient clinics^16^, hemodialysis^19^, perioperative period^15^ and burned patients^28^.

In addition, the study found that the greater the age, the lower is the level of self-perceived state anxiety, corroborating other studies that reported that elderly clients submitted to hemodialysis who have a better *performance status* had lower levels of anxiety^4^. It was understood, therefore, that this sample had a good functional capacity, since there was an inversely proportional relationship between physical functional capacity of elderly patients submitted to hemodialysis treatment and anxiety levels.

A similar negative correlation was found between the mean score of state anxiety in women compared to that observed in men, which also occurred in an international study in which the average anxiety among women (mean = 25.00, SD = 5.59) was higher than that found among men (mean = 21.93, SD = 7.30)^29^. This may be related to concern about family dynamics, requiring studies addressing a naturalistic paradigm in order to better evaluate this phenomenon.

It is noteworthy that in the experimental group, the statistical and clinical reductions of systolic and diastolic blood pressure, heart rate and respiratory rate were perceptible. These reductions corroborate a study carried out with 172 individuals in outpatient surgery who had reduced anxiety and reduced vital parameters in relation to baseline values^14^ and a meta-analysis that aimed to describe the effect of musical intervention in the treatment of hypertension, with results of reduction of systolic blood pressure from 144 mmHg to 134 mmHg and diastolic blood pressure from 84 mmHg to 78 mmHg^30^.

A cohort study conducted in the Netherlands for three years identified that CKD patients with anxiety symptoms showed a trend of greater propensity for adverse events and worse clinical outcome^13^. In this sense, the use of music is in harmony with a more humanized care. Associated knowledge and practical use of musical interventions in the health area give rise to physiological effects and metabolic changes, adrenaline release, regulation of vital parameters, reduction of fatigue, increase of threshold sensory stimuli, besides improvement of cognition. Thus, this intervention can be used as a complementary therapeutic resource in nursing practice^11^ ^-^ ^12^ ^,^ ^22^.

It should be emphasized that the inclusion of music in nursing interventions is not characterized as a practice of music therapy, because this is a competence of music therapists, professionals with mastery of specific therapeutic skills on the use of music and its elements. However, because music is recognized in different national and international studies for its effectiveness in face of various health problems and because it is represented as intervention in documents that regulate the interventionist practices of nurses (NIC), music represents a possible intervention of low cost that can be used in imbalances in the health state.

Some limitations need to be mentioned. The sample was small and, although the study was conducted in three renal replacement therapy clinics, it comes from a single state of the country, thus hindering the external generalization of the findings to other regions of Brazil. We suggest further multicenter studies in different regions of the country.

Another issue was the non-use of music of the patient’s preference, an activity listed in the classification of nursing interventions of the NIC for application of music therapy intervention^10^. The music chosen for the study belongs to a musical genre that is not among the musical preferences of the majority of the Brazilian population, perhaps because of the difficult access to classical productions. This fact does not undermine the choice though, because the objective of this research was to prove the therapeutic effect of music in the reduction of anxiety as based on international studies^12^ ^,^ ^24^ ^)^ which have confirmed the effective results with this musical genre. Moreover, one study corroborates that the use of music of the patient’s preference did not present a statistically significant effect on anxiety when compared to classical music (p = 0.769)^29^.

It is therefore recommended that future studies compare the effect of songs of the patient’s preference with classical music on the reduction of anxiety and vital parameters of patients undergoing renal replacement therapy.

This clinical trial was conducted based on the CONSORT guidelines and for this reason it possible to reproduce this study, with the possibility of using its results in subsequent systematic reviews.

## Conclusion

There was a statistically significant difference between the groups regarding anxiety and vital parameters, demonstrating that musical intervention is a therapeutic resource that can be used in the care provided by nurses, in order to help reduce anxiety and change vital parameters caused by anxiety in chronic renal patients undergoing hemodialysis.

We hope that this study may serve for future applications and that its results stimulate the use of complementary practices by nurses in their daily lives.
